# Chemotherapeutic dihydromyricetin with remarkable anti-tumor activity and biosafety for muscle invasive bladder cancer

**DOI:** 10.3389/fphar.2025.1609354

**Published:** 2025-07-18

**Authors:** Zicheng Guo, Wang Wang, Weikang Hu, Wenjie You, Zijian Wang

**Affiliations:** ^1^Department of Urology, The Central Hospital of Enshi Tujia and Miao Autonomous Prefecture, Enshi, China; ^2^Hubei Key Laboratory of Urological Diseases, Department of Urology, Cancer Precision Diagnosis and Treatment and Translational Medicine Hubei Engineering Research Center, Zhongnan Hospital of Wuhan University, Wuhan, China; ^3^Ministry of Education Key Laboratory of the Green Preparation and Application for Functional Materials, Hubei Key Laboratory of Polymer Materials, School of Materials Science and Engineering, Hubei University, Wuhan, China; ^4^Orthopedic Hospital, Postdoctoral Innovation Practice Base, The First Affiliated Hospital, Jiangxi Medical College, Nanchang University, Nanchang, China

**Keywords:** dihydromyricetin, anti-tumor activity, biosafety, bladder cancer, epithelial-mesenchymal transition

## Abstract

Plant-derived drugs (PDD) with remarkable anti-tumor activity and biosafety are highly desirable for clinical tumor chemotherapy. In this work, dihydromyricetin (DHM), a natural PDD extracted from ratten tea, was screened out to be a potential chemotherapeutic drug for muscle invasive bladder cancer (MIBC). The results of *in vitro* assays confirmed that DHM could effectively inhibit the proliferation, survival and migration of MIBC cells, and promote apoptosis (*P* < 0.05). M1 macrophage polarization was also observed after DHM chemotherapy. The hub genes in cell cycle and apoptosis signaling pathways were differential expressed, and the epithelial-mesenchymal transition (EMT) in MIBC cells was also reversed by DHM treatment. The *in vivo* effectiveness and biosafety evaluations of DHM chemotherapy were performed using a xenograft bearing mice model. The results revealed that DHM intravenous chemotherapy with a dose of 20 mg/kg for 7 times could significantly suppress the *in vivo* tumorigenesis of MIBC (*P* < 0.05), while triggered no obvious drug side effects. In conclusion, this work provided a PPD with remarkable *in vitro* and *in vivo* anti-tumor activity and biosafety, which could serve as a promising alternative for the application of MIBC chemotherapy.

## Introduction

Bladder cancer (BCa) exhibits the highest morbidity and mortality rates among genitourinary malignancies, with approximately 430,000 new cases and 170,000 deaths reported annually (Flaig et al., 2024; Antoni et al., 2017). Based on histopathological characteristics, BCa can be categorized into two distinct groups: muscle-invasive bladder cancer (MIBC), which is typically of high grade, and non-muscle-invasive bladder cancer (NMIBC), which is generally of low grade (Dyrskjot et al., 2023). Clinicians and basic researchers have utilized laparoscopic and da Vinci robot-assisted surgical procedures in the treatment of MIBC (Wang L. et al., 2024; Ai et al., 2024). However, the therapeutic outcomes for MIBC in clinical settings remain suboptimal, with patient survival significantly compromised by the recurrence and metastasis of the cancer (Baack, 2023; Liu G. et al., 2019). An epidemiological investigation indicates that the 5-year mortality rate for patients with MIBC without lymph node metastasis is approximately 18.6%, whereas for those with lymph node metastasis, the rate increases significantly to 77.6% (He et al., 2018; Liu et al., 2025). Therefore, the implementation of postoperative interventions is essential to prevent the recurrence and metastasis of MIBC.

Routine intravenous chemotherapy is one of the most commonly employed postoperative treatments across a wide range of tumors (Cockrell and Rose, 2023; Liu et al., 2023). Powles et al. have published findings from a multicenter, phase III clinical trial investigating the efficacy of altezizumab in combination with platinum-based chemotherapy for the treatment of locally advanced and metastatic urological carcinoma (Powles et al., 2018; Sonpavde and Shariat, 2012; Ma et al., 2024; Xiao et al., 2025). Few drugs and their combinations can completely kill cancer cells *in vivo*, partly owning to the individual differences between MIBC patients. Currently, the study on anti-tumor drug is of increasing interest. Food and Drug Administration (FDA, USA) has approved at least 59 anti-tumor drugs whose response rate (RR) were assessed in more than 100 patients. Thus, we plan to screen a novel candidate drug with enhanced anti-tumor activity and biosafety for MIBC chemotherapy. The available MIBC chemotherapeutic drugs in China are mainly composed of gemcitabine, vincristine, methotrexate and pirarubicin.

Numerous plant-derived drugs (PDD) have been demonstrated to exhibit biocompatibility and non-toxicity for *in vivo* applications (Seca and Pinto, 2018; Abou Chakra et al., 2024). It is estimated that thirteen antitumor PDD, such as paclitaxel, vincristine and colchicine, have obtained the U.S. new drug certificate since 1955 (Qu et al., 2019a; Qu et al., 2019b). Our research group concentrated on investigating innovative chemotherapeutic agents for muscle-invasive bladder cancer (MIBC), including capsaicin, resveratrol, plumbagin, and nicotinamide. We have documented that capsaicin is capable of inhibiting the growth of MIBC xenografts *in vivo* through mechanisms involving FOXO3a-mediated signaling pathways (Qian et al., 2016). Numerous Chinese herbal remedies with potential anti-tumor properties are documented in traditional medical literature, including the “Compendium of Materia Medica” (Seca and Pinto, 2018). In recent decades, some anti-tumor components have been extracted from crude drugs by chemical methods, and started next-generation of drug screening (Zhang et al., 2019; Huang et al., 2024).

Dihydromyricetin (DHM), also known as ampelopsis, is a naturally occurring flavonoid derived from plants (He et al., 2025). DHM can be extracted from the smashed ratten tea by distillation and vacuum crystallization, and then recrystallized in acetone solution. Similar to other flavonoids, DHM exhibits a range of pharmacological activities, including anti-tumor, cardioprotective, anti-diabetic, and neuroprotective effects, among others (Zhao et al., 2014; Wang et al., 2023). Particularly, the anti-tumor function and mechanism of DHM has been investigated among lung cancer, ovarian cancer, colorectal cancer and triple-negative breast cancer (Fan et al., 2017; Wang et al., 2019; Wang et al., 2017). Xu et al. reported that DHM significantly induces apoptosis in ovarian cancer cells and effectively reverses chemotherapy resistance mediated by the p53 gene (Xu et al., 2017). Tieng et al. reported that DHM could inhibit the invasion of the triple negative breast cancer by blocking extracellular matrix (ECM) degradation (TiengAmpelopsin et al., 2019). Furthermore, our prior research identified the inhibitory effects of DHM on the proliferation of BCa cells. Nevertheless, the application of DHM in the treatment of MIBC has been scarcely explored, and its anti-tumor properties and underlying mechanisms remain to be elucidated.

In this work, DHM has been chosen as the prospective chemotherapeutic agent for the treatment of MIBC. The anti-tumor efficacy and biosafety profile of DHM against MIBC cells are systematically evaluated through a series of *in vitro* and *in vivo* experiments, including MTT assays, cell migration assays, flow cytometry, Western blotting, and histopathological analyses. It is assumed that DHM can inhibit the tumorigenesis of MIBC without causing obvious drug toxicity. This work will screen out a prospective chemotherapeutic drug, which can be used alone in practice, or further processed into nanomaterials for MIBC precise treatment.

## Materials and methods

### Reagents and chemicals

Commercial dihydromyricelin (DHM, C_15_H_12_O_8_, analytical purity, CAS: 27,200–12–0) was purchased from Macklin Biochemical Co., Ltd. (Shanghai, China). DHM was dissolved into dimethyl sulfoxide (DMSO) to obtain a stock solution with a concentration of 200 mM, and stored at −20°C for further applications. DMSO, absolute ethanol, methanol, crystal violet, paraformaldehyde (PFA) and paraffin were obtained from Sinopharm Chemical Reagent Co., Ltd. (Shanghai, China). RPMI-1640 and DMEM high glucose medium, fetal bovine serum (FBS), penicillin, streptomycin, and trypsin were purchased from Thermofisher Scientific Co., Ltd. (Waltham, MA, USA). The other chemical and biological reagents were used as received.

### Bladder cancer cell lines

The muscle invasive bladder cancer (MIBC) cell lines (T24 and UMUC3) were purchased from the American Type Culture Collection (ATCC) (Manassas, VA, USA). T24 cell was maintained in RPMI-1640 complete medium supplied with 10% FBS and 100 U/mL penicillin/streptomycin solution. UMUC3 cell was maintained in DMEM high glucose complete medium containing FBS and antibiotics. T24 and UMUC3 cells were both cultured using a 37°C humid incubator with 5% CO_2_.

### Cell viability and proliferation assay

First, DHM stock solution with a concentration of 200 mM was diluted using complete medium until the desired DHM concentration (0, 5, 10, 20, 30 μM) was achieved which was named DHM work solution.

In this study, MTT assay was performed to determine the appropriate DHM drug concentration for further *in vitro* evaluations. Briefly, T24 or UMUC3 cells were digested using 0.25% trypsin solution, and centrifuged at 1,200 rpm for 5 min. Then adjusted cell concentration to 1.5 × 10^4^ cells/mL and 200 μL cell suspension was added into each well of 96 well plates. After incubated at 37°C for 6 h, the culture medium was removed and the DHM working solution (0, 5, 10, 20, 30 μM) were added. After incubated at 37°C for 48 h 20 μL MTT reagent was added, followed by incubation for another 4 h. After that, all liquids in the 96 plates were thoroughly wiped out, and 150 μL DMSO was added into each well. The value of optical density (OD) at a wavelength of 490 nm was detected using a microplate reader (SpectraMax^@^M2, MD, USA). The relative cell viability and 50% inhibiting concentration (IC50) were calculated (You et al., 2025).

To further assess the proliferation ability of MIBC cells, DHM working solution (0, 5, 20 μM) was incubated T24 and UMUC3 cells with for successive 5 days 3.0 × 10^3^ cells T24 and UMUC3 cells were added into each well of 96 well plates and DHM working solution were added. Five identical samples were prepared for each group. At regular time intervals (every 24 h), the treated cells (one of the five 96 well plates) were taken out of the incubator, and the value of OD490 was detected. Six independent samples were calculated for statistical comparison.

### Clonogenic survival assay

T24 and UMUC3 cells were collected and re-suspended into DHM working solutions (0, 5 and 20 μM). These cells were seeded onto 6 well tissue culture plates with cell density of 800 cells/well, and then cultured for 12 days 4% paraformaldehyde (PFA) solution was added to fix the cells. After that, 1 mL 0.1% crystal violet solution was applied to visualize the cell clone. The images of cell clones were captured using a digital camera (A7RⅢ, Sony, Japan), and the number of cell clones was counted from at least three independent samples.

### Transwell chamber assay

In this work, transwell chambers (Millipore, Corning, USA) were used to investigate the migration ability of MIBC cells. Briefly, DHM stock solution (200 mM) was diluted using FBS-free basic medium to 0, 5, 20 μM, respectively. The obtained solutions were suspended with T24 or UMUC3 cells with a concentration 4 × 10^5^ cells/mL. 0.1 mL cell suspension was added into the upper layer of transwell chamber, and 0.7 mL complete medium was added into the lower layer. After incubated at 37°C for 24 h, the un-migrated cells on the upper chamber layer were removed carefully using a cotton swab. The migrated cells were fixed with 4% PFA solution for 30 min, and then stained with 0.1% crystal violet solution for 15 min. The cell images were captured by an inverted fluorescence microscope (IX73, OLYMPUS, Japan), the IPP-6.0 software was used for quantitative analysis.

### Wound healing assay

T24 and UMUC3 cells were seeded onto 6 well tissue culture plates with high cell density, and then cultured overnight until the cell confluence reached to 80%. A linear cell wound was created using 200 μL pipette tips. After washed twice with PBS solution, the culture plates were added with DHM working solutions with a concentration of 0, 5, 20 μM, respectively. Before and after 24 h of incubation, the morphology of cell wounds was captured using an inverted fluorescence microscope (IX73, OLYMPUS, Japan). The wound healing rate was calculated as follows:

$$\text{Wound healing rate }\left(\%\right)=\left(W0-W24\right)/W0*100$$

Where W0 and W24 corresponded to the width of cell wounds before and after 24 h of incubation, respectively.

### Flow cytometric analysis

#### Cell cycle

The cell cycle analysis was carried out in accordance with our precious report (Ge et al., 2019). T24 and UMUC3 cells were co-incubated with DHM working solutions (0, 5, 20 μM) for 48 h, and then collected into 1.5 mL centrifuge tubes. The obtained cells were washed using PBS solution for three times. Cell cycle staining kit was purchased from Multi Sciences Co., Ltd. (Hangzhou, China). 1 mL DNA staining solution and 10 μL permeabilization solution was added into each tube. After incubated in the dark place for 30 min, the cell cycle was detected using a flow cytometry (CytoFLEX, Beckman, USA). At least three independent samples were tested in each group.

### Cell apoptosis

MIBC cells were treated with DHM working solutions (0, 5, 20 μM) for 48 h, and then collected into 1.5 mL tubes for apoptosis staining. The Annexin V-FITC/PI apoptosis kit was obtained from BD Biosciecnes Co., Ltd. (San Jose, USA). The DHM treated cells were re-suspended in the 1 × binding buffer, and then stained with Annexin V-FITC for 15 min and PI for another 10 min. The apoptosis rate of all samples was detected using a flow cytometry (CytoFLEX, Beckman, USA). At least three independent samples were tested in each group.

### Phenotypic characterization

T24 cells were incubated with DHM working solutions (0, 5, 20 μM) for 12 h, and the supernatant was collected for phenotypic characterization. Human monocytic leukemia cell line (THP-1) was seeded in 6 well tissue culture plates with a density of 1 × 10^6^ cells/well. After incubated with the supernatant for 24 h, the THP-1 cells were collected and treated with CD11b/c-APC, CD86-FITC, CD80-PE. The stained cells were then analyzed using a flow cytometry (CytoFLEX, Beckman, USA). The percentage of positive cells indicated the expression degree of the surface markers, and calculated from three independent samples (Liu et al., 2020).

### Quantitative real-time PCR

The total RNA molecules from the treated MIBC cells were isolated using a HiPure RNA Mini Kit (Magen, China). The RNA concentration was then measured using an ultraviolet spectrophotometer (Nanodrop 2000, Thermo, USA). The reverse transcription reaction was carried out, and the obtained cDNA was used for quantitative real-time PCR (qRT-PCR). The iQTM SYBR®Green Supermix was purchased from Bio-Rad Co., Ltd. (Shanghai, China) and applied for qRT-PCR. Primer sequences used in this work are listed in Supplementary Table S1.

### Western blot and immunofluorescence (IF) staining

T24 and UMUC3 cells were incubated with DHM working solutions (0, 5, 20 μM) for 48 h before they were harvested for Western blot analysis. The treated cells were completely lysed in 1 mL RIPA buffer containing 20 μL phosphatase and protease inhibitor mixture. The lysis supernatant was collected after centrifuged at 1.2 × 10^4^ g for at least 15 min. The total protein concentration was evaluated using a BCA protein assay kit (Abcam, China). The protein samples were denatured and separated by 7.5%–15% SDS - PAGE gels, and then transferred to a PVDF membrane (Millipore, USA). The PVDF membranes were blocked using 5% fat-free milk for 2 h and incubated with primary antibodies overnight and secondary antibodies for 2 h. The primary and secondary antibodies used in this work are listed in Supplementary Table S1. The enhanced chemiluminescence (ECL) kit was purchased from BD Biosciecnes Co., Ltd. (San Jose, USA). The protein bands were detected using a ChemiDoc™ MP Imaging System (Bio-Rad, USA). IF staining of Ki67 protein were performed according to the general protocols, and accomplished by Biofavor Biotech Co., Ltd. (Wuhan, China). The cell images of IF staining were captured by an inverted fluorescence microscope (IX73, OLYMPUS, Japan).

### *In vivo* xenograft experiments

This work was carried out in accordance with the declaration of Helsinki. Six BALB/C57 nude mice, aging about 3 weeks, were purchased from WQJX Bio-Technology Co., Ltd. (Wuhan, China). These animals were quarantined in SPF experimental facility for 7 days to adapt the new environment. The ethics committee at Zhongnan Hospital of Wuhan University has approved this research.

200 μL T24 cells (1.5 × 10^7^ cells/mL in PBS) were subcutaneously injected into the mice. The animals were fed for 15 days before DHM drug injection. DHM was firstly dissolved in absolute alcohol with a concentration of 10 mg/mL, and then diluted using normal saline to prepare the DHM working solution. DHM working solution was injected at a dose of 20 mg/kg every third day for 21 days. For blank control, animals were injected with normal saline containing equivalent alcohol. Tumor size of the treated animals was measured using a vernier caliper, and then used to calculate the tumor volume (mm^3^) as A_L_ × A_W_^2^× 0.5, where A_L_ and A_W_ refers to the length and width of the tumors, respectively. After sacrificing the animals, all tumor samples, blood samples and organs samples including heart, liver, spleen, lung, kidney and brain, were harvested for further tests.

The tumor samples and organs were fixed with 4% paraformaldehyde (PFA) solution for 48 h. These samples were embedded by paraffin and then sliced into 4 μm sections. Hematoxylin-eosin (HE) and Masson’s trichrome (MT) staining, immunofluorescence (IF) staining of Ki67, and TUNEL assay were performed according to the general protocols. An inverted fluorescence microscope (IX73, OLYMPUS, Japan) was used for data acquisition.

The concentration of alanine aminotransferase (ALT), aspartate aminotransferase (AST), γ-glutamyl transpeptidase (GGT), creatinine (CRE), and total bilirubin (TBIL) was detected using an enzyme linked immunosorbent assay (ELISA). Commercial ALT, AST and GGT ELISA kits were purchased from BOSTER Biotechnology Co., Ltd. (Wuhan, China). CRE and TBIL ELISA kits were purchased from Beyotime Biotechnology Co., Ltd. (Shanghai, China). The blood samples were centrifuged at 3,000 rpm for 5 min, and then diluted using distilled water. ELISA test was performed according to the manufacturers’ protocols. At least three samples were used for statistical analysis.

### Statistical analysis

The data from no less than three independent biological evaluations were expressed as mean ± standard deviation (SD). One-way ANOVA as well as *post hoc* Tukey’s test was applied for statistical analysis. *P* < 0.05 was considered to be statistically different.

## Results

### DHM concentration for *in vitro* evaluations

**FIGURE 1:**
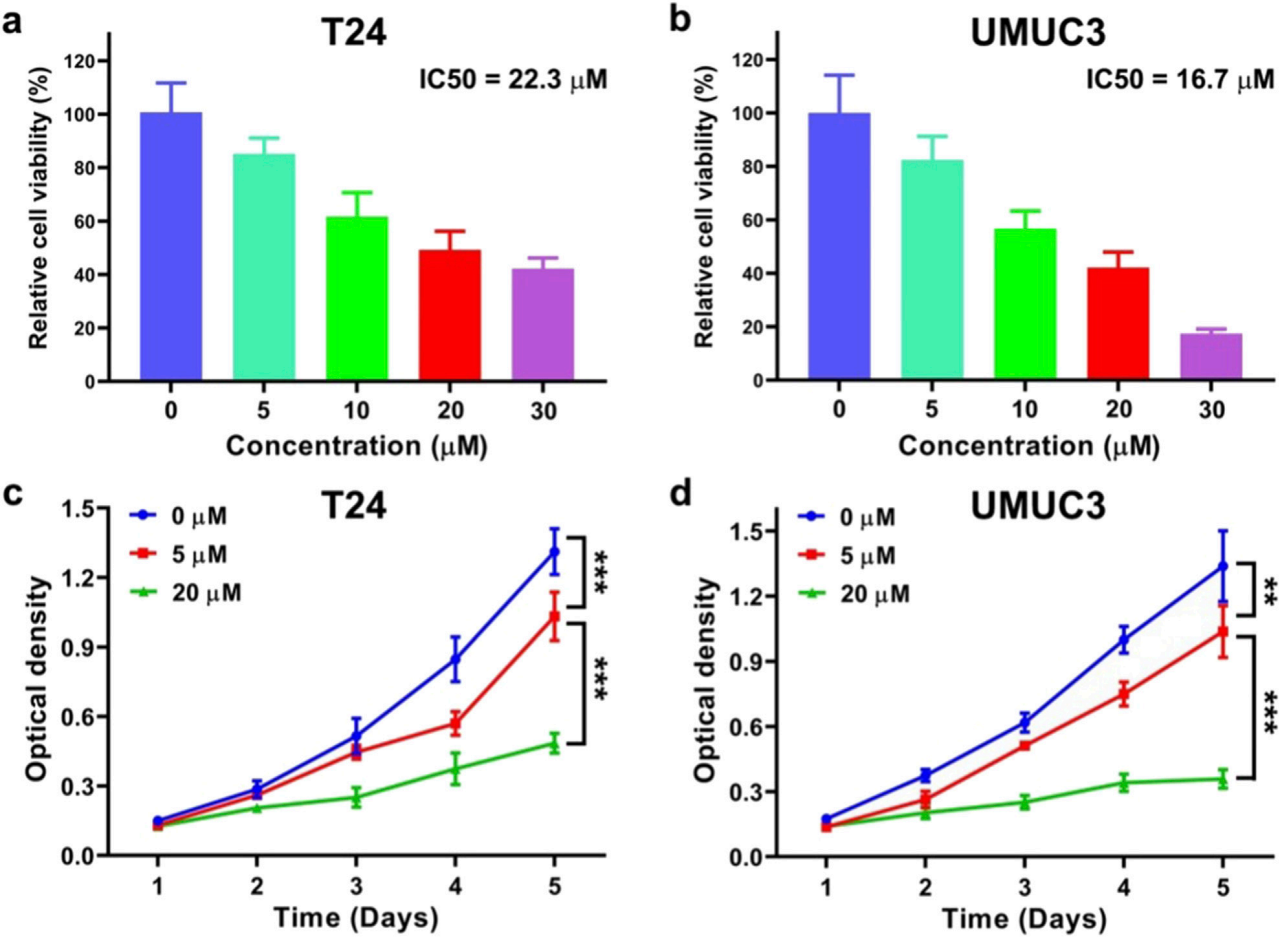
Dihydromyricetin (DHM) inhibited the viability and proliferation of MIBC (T24 and UMUC3) cells *in vitro*. **(a,b)** To determine the appropriate DHM concentration for *in vitro* tests, T24 and UMUC3 cells were co-incubated with DHM at a concentration of 0, 5, 10, 20 and 30 μΜ, respectively. The relatively cell viability were assessed by MTT assay, and the value of 50% inhibiting concentration (IC50) was calculated; **(c,d)** The MTT proliferation curves of T24 and UMUC3 cells which were incubated with DHM at 0, 5 and 20 μM for consecutive 5 days **P < 0.01, ***P < 0.001.

To screen out the appropriate DHM drug concentration for *in vitro* experiments, T24 and UMUC3 cells were co-incubated with DHM solution (0, 5, 10, 20 and 30 μM), and the relative viability of treated cells was assessed by MTT assay. As shown in Figures 1a,b, the relative cell viability after DHM treatment decreased significantly along with an increase of DHM concentration from 0 to 30 μM, suggesting that DHM possessed the potential anti-tumor activity against MIBC. The IC50 values of DHM were 22.3 μM for T24 cell, and 16.7 μM for UMUC3 cell. Thus, we intended to set three independent groups in the following experiments: high dose group (20 μM), low dose group (5 μM) and blank control group (0 μM).

### DHM inhibited MIBC cell proliferation in a dose-dependent manner

MTT assay was also applied to evaluate the anti-proliferation activity of DHM against MIBC cells. The proliferation curves of T24 and UMUC3 cells are shown in Figures 1c,d. The optical density (OD) value declined significantly as the DHM concentration increased. At the fifth day, the OD values of T24 cell were 1.31 ± 0.10 for 0 μM group, 1.03 ± 0.10 for 5 μM group and 0.49 ± 0.04 for 20 μM group. Meanwhile, the OD values of UMUC3 cell were 1.34 ± 0.16 for 0 μM group, 1.03 ± 0.12 for 5 μM group and 0.36 ± 0.04 for 20 μM group. Significant difference was observed between each group (*P* < 0.05). In conclusion, DHM possessed a dose-dependent anti-proliferation activity against MIBC cells.

### DHM induced cell cycle arrest

**FIGURE 2:**
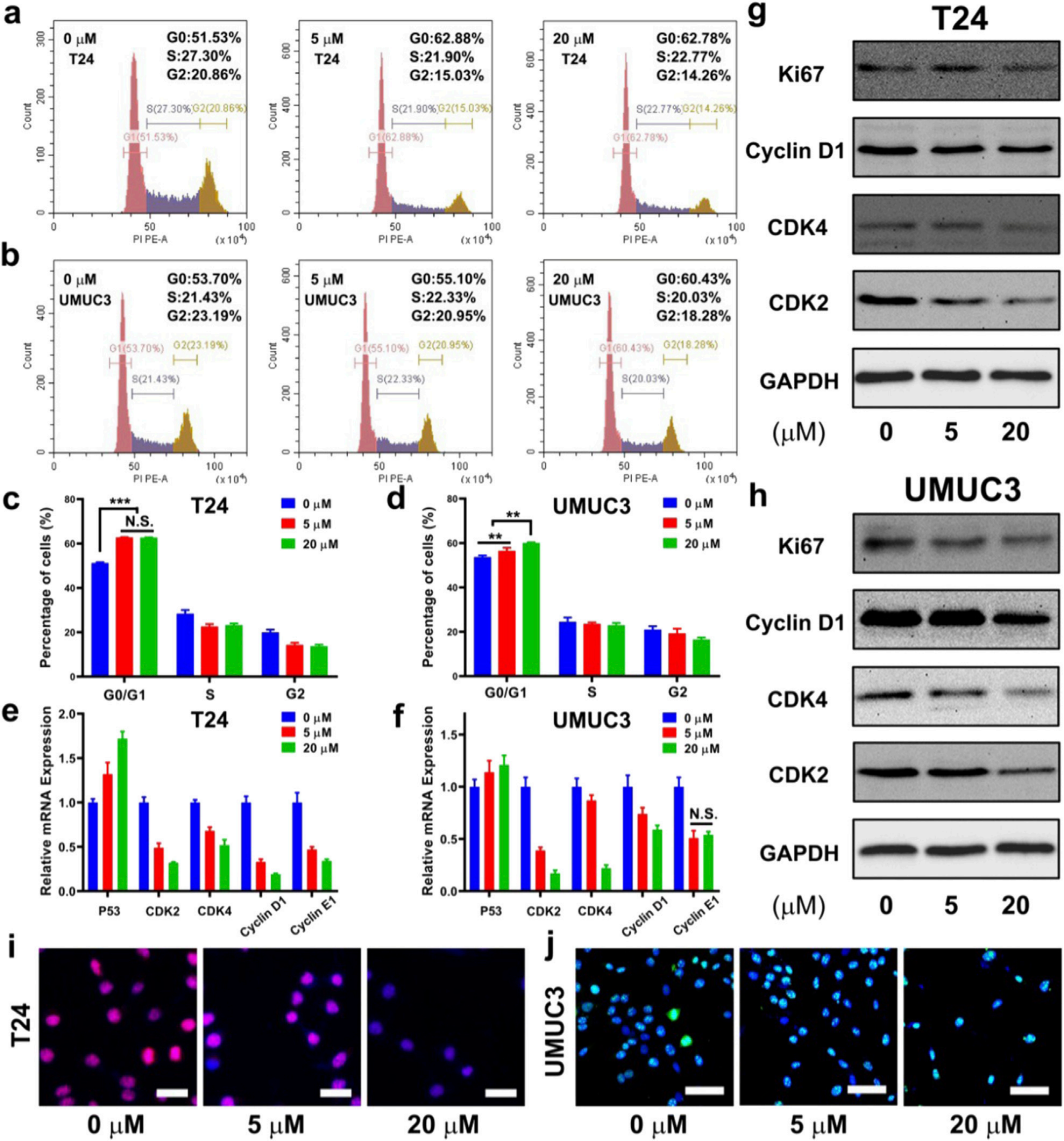
Dihydromyricetin (DHM) induced MIBC (T24 and UMUC3) cells cycle arrested in G0/G1 phase. **(a,b)** Representative flow cytometry images of cell cycle in MIBC cells after treated with DHM for 48 h; **(c,d)** Quantitative results of cell cycle distribution exhibited a significantly increase in G0/G1 phase cell percentages. **P < 0.01, ***P < 0.001; **(e,f)** The relative expression of cell cycle related genes (P53, CDK2, CDK4, Cyclin D1, Cyclin E1) in both kinds of cells could be modulated by DHM; **(g,h)** Western blot images revealed an obvious downregulation of cell cycle proteins, such as CDK2, CDK4, Cyclin D1 and Ki67; **(i,j)** Representative immunofluorescence (IF) images of Ki67 staining. Blue signal (DAPI): cell nucleus. Red/green signal: Ki67 protein. Scale bar: 100 μm.

To investigate the mechanism of anti-proliferation activity of DHM, cell cycle analysis, qRT-PCR and Western blot assay were performed. The results of cell cycle analysis are shown in Figures 2a–d, the percentage of MIBC cells in G0/G1 phase was significantly increased after DHM treatment (*P* < 0.05). For T24 cell, the percentage of G0/G1 phase cells increased from 51.3% ± 0.4% (0 μM group) to 62.8% ± 0.1% (5 μM group) and 62.7% ± 0.1% (20 μM group). For UMUC3 cell, the percentage of G0/G1 phase cells increased from 53.7% ± 0.7% (0 μM group) to 56.5% ± 1.3% (5 μM group) and 60.1% ± 0.3% (20 μM group). This phenomenon suggested that DHM could effectively induce cell cycle arrest, which played a vital role in tumor development. The expression level of cell cycle related genes (*P53*, *CDK2*, *CDK4*, *Cyclin D1* and *Cyclin E1*) was tested by qRT-PCR assay. As shown in Figures 2e,f, P53 gene in the 5 μM and 20 μM groups was upregulated while the other genes were significantly downregulated (*P* < 0.05). The results of Western blot also exhibited an obvious downregulation of CDK2, CDK4, Cyclin D1 and Ki67 proteins in both T24 and UMUC3 cells (Supplementary Figures S2g, h; Supplementary Figures S4a–c). CDK2/4 and Cyclin D1/E1 can form the hub complexes to participate in multiple biological signaling pathways, and drive cell cycle from G0/G1 phase to S phase. Thus, the abnormal expression of these genes might interrupt the cell cycle pathway, and eventually lead to the proliferation inhibition effect among MIBC cells. Ki67 is one of the markers for cell proliferation activation state. As shown in Figures 2i,j, fluorescence intensity (FSI) of the T24 and UMUC3 cells was both faded away after DHM treatment. This phenomenon has further confirmed that DHM could inhibit MIBC cells proliferation, which was consistent with the results in Figures 1, 2.

### DHM activated the apoptosis pathway

**FIGURE 3:**
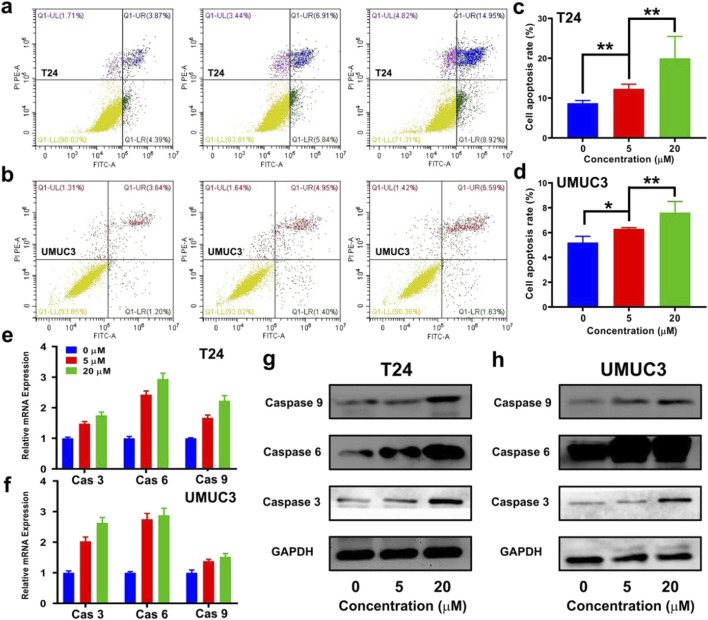
Dihydromyricetin (DHM) promoted MIBC (T24 and UMUC3) cells apoptosis. **(a,b)** Representative flow cytometry photos of apoptosis in T24 and UMUC3 cells after treated with DHM for 48 h; **(c,d)** Quantitative results of cell apoptosis rate exhibited an effectively pro-apoptotic activity of DHM. *P < 0.05, **P < 0.01; **(e,f)** The relative expression of Caspase 3/6/9 in T24 and UMUC3 cells after DHM treatment; **(g,h)** Western blot images revealed a strong upregulation of apoptosis proteins.

The cell apoptosis was evaluated using a Annexin V-FITC/PI staining method. The representative images of flow cytometry are shown in Figures 3a,b, and its quantitative results are shown in Figures 3c,d. After co-incubated with DHM for 48 h, the apoptosis rate of T24 and UMUC3 cells were both significantly improved (*P* < 0.05). For T24 cell, the cell apoptosis rate increased from 8.7% ± 0.7% (0 μM group) to 12.3% ± 1.1% (5 μM group) and 19.9% ± 5.5% (20 μM group). For UMUC3 cell, the cell apoptosis increased from 5.2% ± 0.5% (0 μM group) to 6.3% ± 0.1% (5 μM group) and 7.6% ± 0.9% (20 μM group). Apoptosis is an independent and programmed way of death, which is regulated by many genes like caspase 3/6/9. Particularly, caspase 3 is the most important protein-cutting enzyme in apoptosis pathway. As shown in Figures 3e,f, the relative mRNA expression of *caspase 3*, *caspase 6* and *caspase 9* in T24 and UMUC3 cells were significantly upregulated by DHM treatment (P < 0.05). Furthermore, the results of Western blot analysis also exhibited an obvious growth trend in caspase 3/6/9 proteins (Figures 3g,h; Supplementary Figures S4d, e). In conclusion, DHM could promote MIBC cells apoptosis via activating the transcription and translation of *caspase 3/6/9*.

### DHM induced the polarization of M1 macrophages

The synergistic effects of tumor killing and immune response was evaluated by flow cytometry. CD80 and CD86 are the markers of M1 macrophages, and CD11b/c is the marker of monocytes. The polarization from initial monocytes to M1 macrophages could be used to indicate immune activation during chemotherapy. The results of CD80 staining are shown in Supplementary Figures S1a, b. The percentage of CD80 positively stained cells increased greatly after DHM treatment (*P* < 0.05). The percentage of CD80 positively stained cells was 0.51% ± 0.48% in the 0 μM group, and then decreased to 1.52% ± 0.4% and 32.1% ± 4.1% in the 5 μM group and 20 μM group, respectively. The results of CD86 staining are shown in Supplementary Figures S1c, d, and exhibited the similar growth trend. The polarization from monocytes to M1 macrophages could be motivated by foreign antigens such as cell debris. In this study, DHM killed MIBC cells, resulting in increased cell debris, and eventually led to M1 macrophage polarization.

### DHM blocked the survival and migration of MIBC cells

**FIGURE 4:**
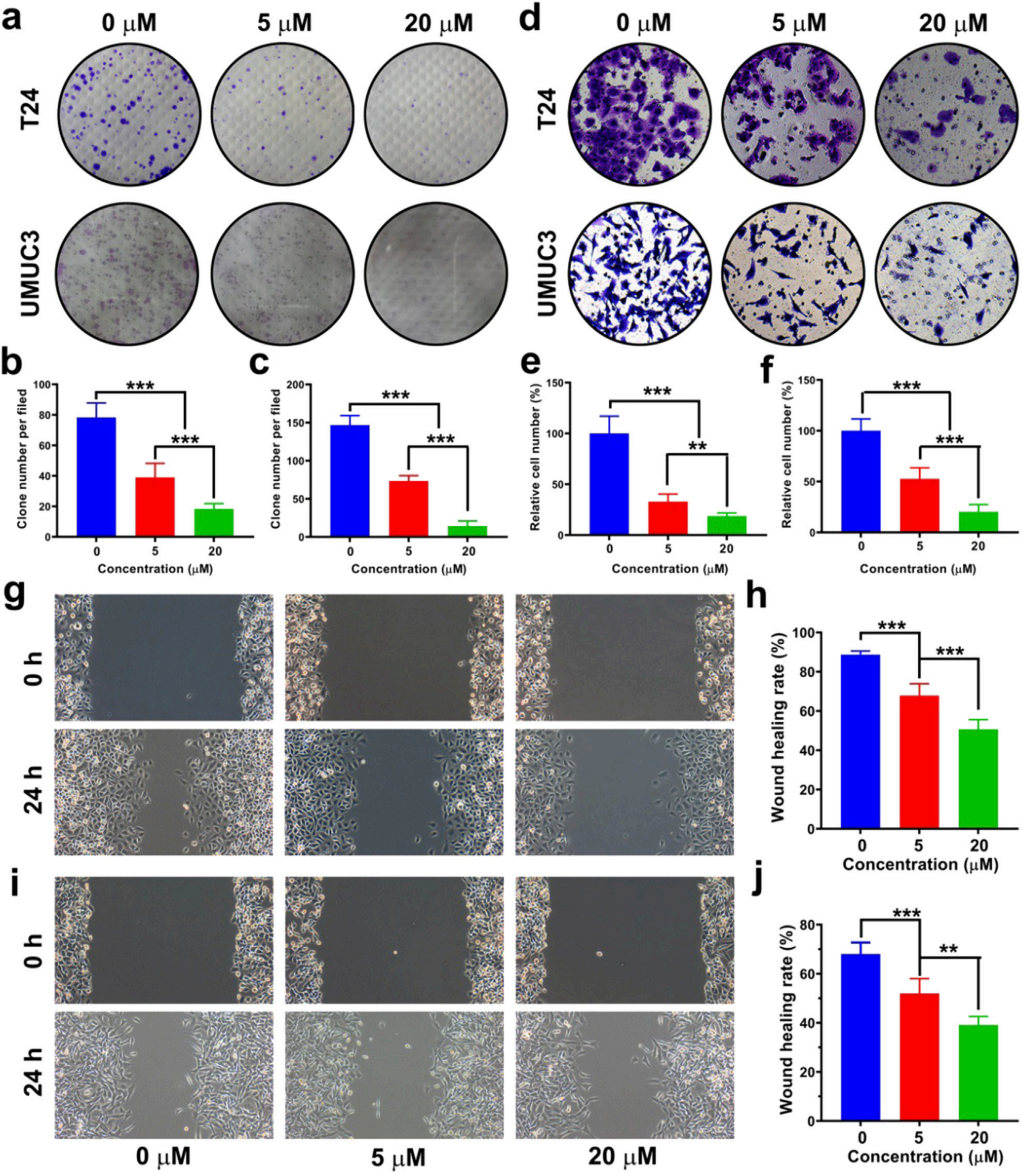
Dihydromyricetin (DHM) inhibited the migration and survival of MIBC (T24 and UMUC3) cells *in vitro*. **(a)** The influence of DHM on MIBC cell survival was evaluated by clonogenic survival assay; **(b,c)** Quantitative results of cell clone number per filed. ***P < 0.001; **(d)** Representative images of transwell chamber assay after DHM treatment. **(e,f)** Transwell chamber assay indicated that the migration ability of T24 and UMUC3 cells was significantly inhibited by DHM. **P < 0.01, ***P < 0.001; **(g,h)** The anti-migration activity of DHM against T24 cell was investigated by wound healing assay; **(i,j)** The anti-migration activity of DHM against UMUC3 cell. **P < 0.01, ***P < 0.001.

In this work, clonogenic survival assay was adopted to investigate the survival ability of MIBC cells as reported before (Zhou et al., 2019). The images of T24 and UMUC3 cell clones are shown in Figure 4a, and the quantitative results of clone number are shown in Figures 3b–d. The clone number of T24 cells was 78.3 ± 9.5 in the 0 μM group, and then decreased to 39.0 ± 9.2 and 18.3 ± 3.5 in the 5 μM group and 20 μM group, respectively. For UMUC3 cell, the clone number was 147.7 ± 12.5 in the 0 μM group, and then decreased to 73.4 ± 7.1 and 14.4 ± 6.5 in the 5 μM group and 20 μM group, respectively. It could be concluded that DHM inhibited BCa cell survival significantly (*P* < 0.05).

Transwell chamber assay and wound healing assay were both performed to evaluate the migration ability of MIBC cells. The representative images in transwell chamber assay are shown in Figure 4d. The migrated cells at the lower layer of transwell chamber were photographed and counted. The relative number of migrated T24 or UMUC3 cells was both declined after DHM treatment (Figures 4e,f). The representative images of T24 and UMUC3 cells in wound healing assay are shown in Figures 4g–i, and the corresponding statistical results of wound healing rate are shown in Figures 4h–j. The result of wound healing assay was in consistent with that of transwell chamber assay. In conclusion, the survival and migration abilities of MIBC cells could be effectively blocked by DHM treatment.

### DHM inhibited epithelial-mesenchymal transition (EMT) in MIBC cells

**FIGURE 5:**
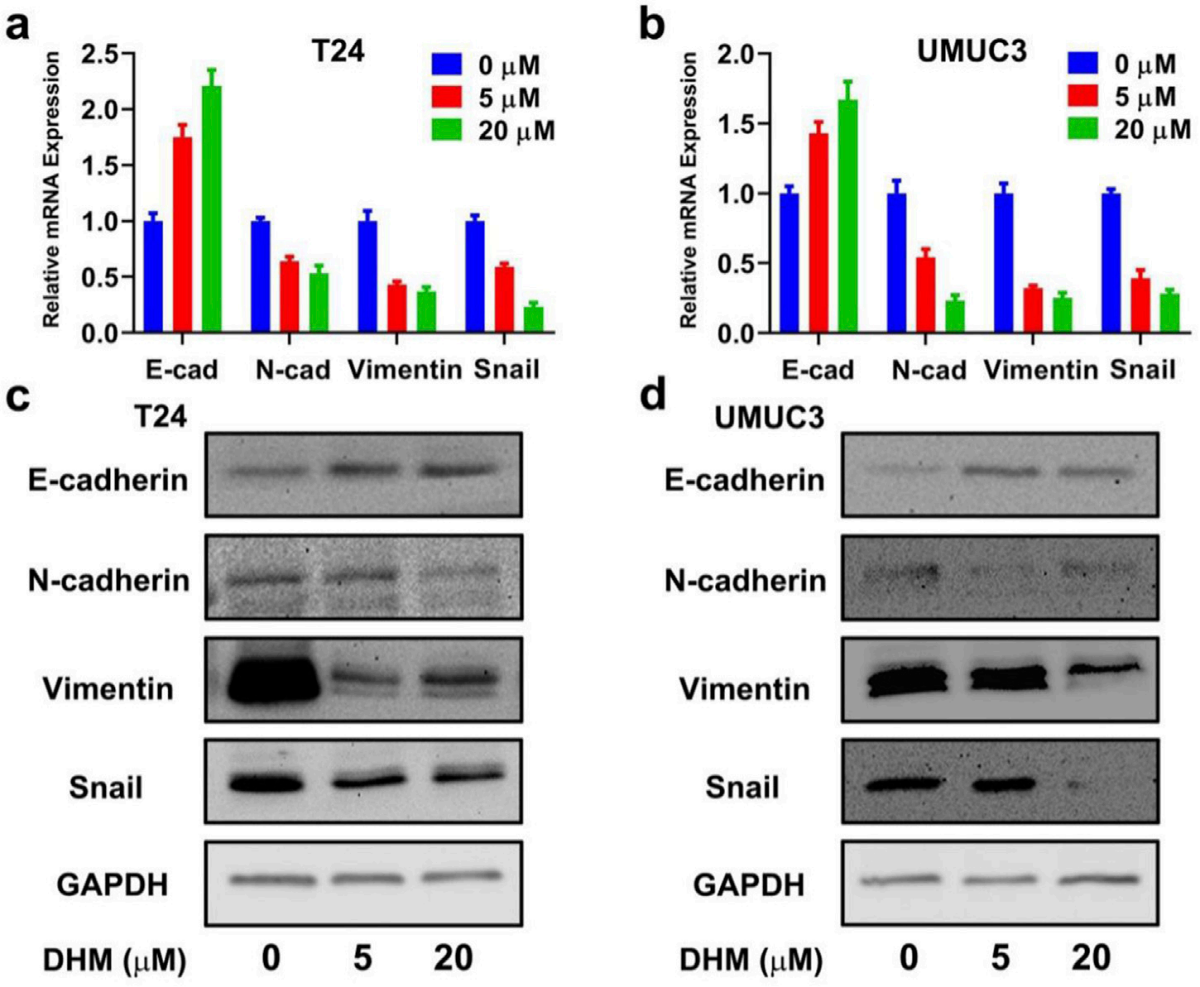
Epithelial-mesenchymal transition (EMT) pathway was blocked by DHM to inhibit MIBC cells migration. **(a,b)** The relative gene expression of EMT bio-markers (E-cad, N-cad, Vimentin, Snail) in T24 and UMUC3 cells, respectively; **(c,d)** E-cad protein expression was upregulated, N-cad, Vimentin and Snail protein expression was downregulated. These results indicated that DHM could block EMT pathway, and then cause the migration inhibition of MIBC cells directly.

DHM can effectively inhibit *in vitro* migration of MIBC cells, but its’ mechanism remains to be unknown. EMT is an important biological process for tumor cells to gain the phenotypic characteristics and metastatic potential of mesenchymal cells. Thus, we intended to investigate the expression of EMT markers, so as to uncover the mechanism of migration inhibition. Mesenchymal markers (N-cad, Vimentin and Snail) and epithelial marker (E-cad) were chosen for expression analysis. As shown in Figures 5a,b, the relative expression of E-cad gene in both T24 and UMUC3 cells were significantly upregulated (*P* < 0.05), while that of N-cad, Vimentin and Snail genes were all significantly downregulated by DHM treatment (*P* < 0.05). The results of Western blot also exhibited similar tendency in both T24 and UMUC3 cells (Figures 5c,d; Supplementary Figures S4g–j). Generally, the EMT process in MIBC cells was successfully inhibited by DHM treatment, which might lead to migration inhibition eventually.

### DHM suppressed *in vivo* tumor growth with biosafety

A xenograft mouse model was created by subcutaneously transplanting T24 cells into the treated animals. The DHM working solution and the blank control solution were prepared and injected intravenously to imitate clinical chemotherapy. The dose of DHM for *in vivo* application was set to be 20 mg/kg, and the frequency of injection was set to be every third day. After 21 days of chemotherapy, the tumor samples were collected for histological analysis. Blood samples and main organs (heart, liver, spleen, lung, kidney, and brain) of the animals were also harvested for *in vivo* biosafety evaluations.

**FIGURE 6:**
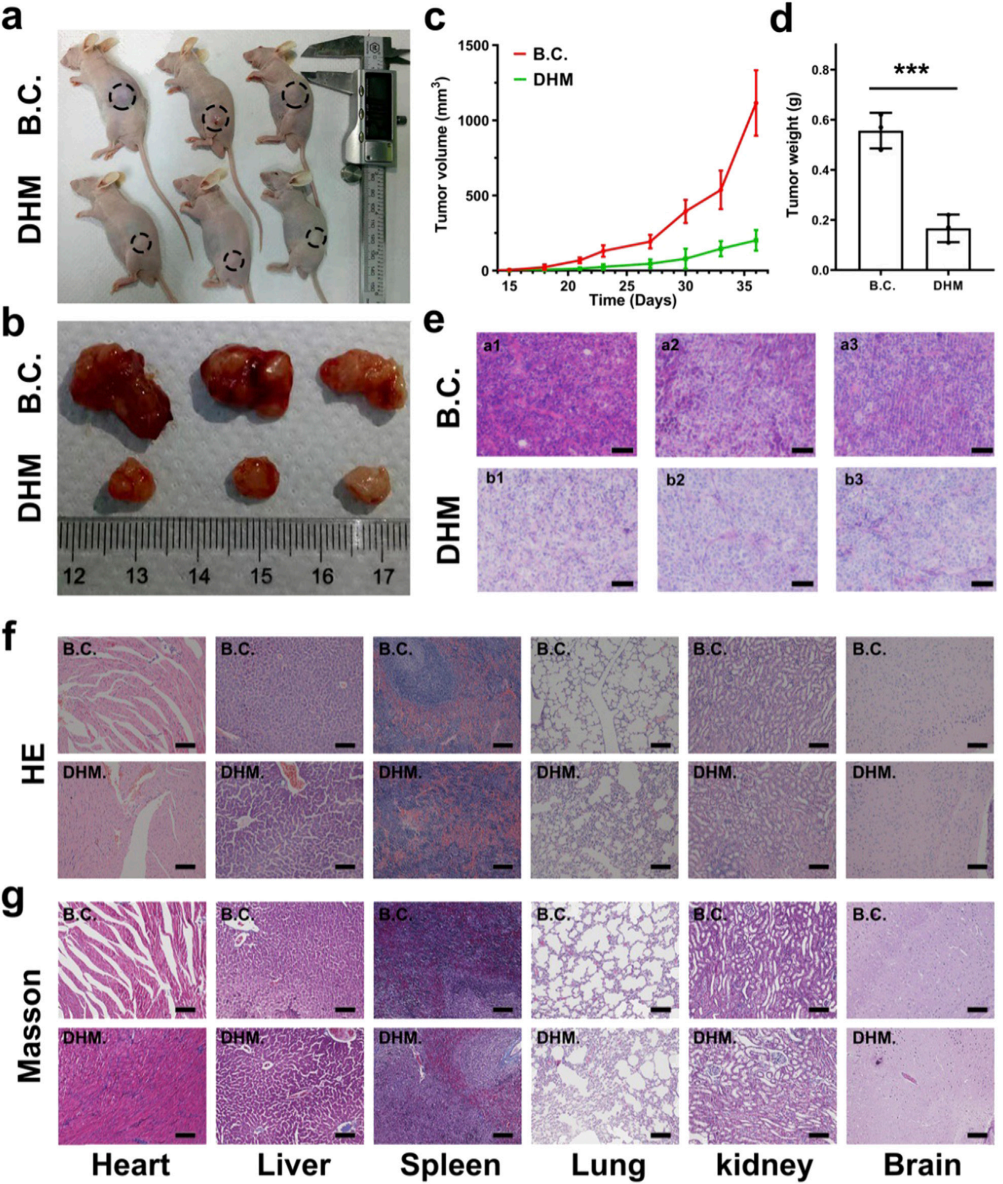
Dihydromyricetin (DHM) suppressed *in vivo* tumorigenesis with good biosafety. **(a)** BALB/c mice were seeded with T24 cell subcutaneously for 15 days. DHM and the control were then injected every third day for additional 21 days; **(b)** The photographs of tumor samples dissected from mice; **(c)** The growth curves of tumor volume from 15 th to 36 th day; **(d)** Quantitative results of tumor weight. ***P < 0.001; **(e)** HE staining images of the tumor samples, a1-3 and b1-3 corresponding to the three individual animals in the DHM group and control groups, respectively. Scale Bar: 100 μm; **(f,g)** Representative HE and Masson staining images of the organs (heart, liver, spleen, lung, kidney and brain) dissected from the treated mice. The results confirmed relatively good *in vivo* biosafety. Scale Bar: 100 μm.

The object image of tumor bearing mice is shown in Figure 6a, and the photo of tumor samples dissected in the 36th day is shown in Figure 6b. It was observed that the tumor volume in the DHM group grew much lower than that in the blank control group (Figure 6c). Compared with blank control, the average tumor weight has decreased from 0.56 ± 0.07 g to 0.17 ± 0.06 g (Figure 6d), significant difference was observed (*P* < 0.001). The HE staining of tumor samples from three pairs of treated animals also confirmed a definitive anti-tumor activity of DHM against MIBC (Figure 6e).

*In vivo* proliferation and apoptosis of the treated MIBC xenografts were further evaluated by immunofluorescence staining and TUNEL assay. As shown in Supplementary Figures S2a, Ki67 protein was dyed red and located in the nucleus, and less Ki67 positively stained cells were found in DHM group. Ki67 is a member of cell cycle related proteins, and its’ upregulation usually leads to rapid proliferation. This result suggested that the proliferation ability of the MIBC xenografts was inhibited by DHM treatment. The images of TUNEL assay are shown in Supplementary Figures S2b. More positively stained cells appeared in DHM group, further indicating that the apoptosis of MIBC xenografts was motivated by DHM treatment.

*In vivo* biosafety of the DHM chemotherapy was evaluated using a histological method. The HE and Masson staining of the organ samples were performed, and the corresponding images are shown in Figures 6f,g. Compared with blank control, the histological structures in DHM group were slightly changed. Typically, pulmonary edema and pulmonary interstitial dilatation were observed. Besides, cardiac myocytes are enlarged and disorganized, which could be attributed to pulmonary cardiac compensatory hyperplasia. Mild inflammatory infiltration was found in heart, lung and kidney. These results suggested that DHM had potential organ toxicity, mainly targeting lung tissue. It is worth mentioning that the toxicity of DHM may be caused by overdose, and the ideal dose of DHM needs to be optimized in subsequent experiments. *In vivo* hemocompatibility of the DHM chemotherapy was also carried out as reported before (Zheng et al., 2019). The blood biochemical indicators, such as ALT, AST, GGT, CRE and TBIL, were detected using an enzyme linked immunosorbent assay (ELISA). The corresponding results are shown in Supplementary Figures S3a, b. Compared with blank control, there was no significant difference in each indicator of the DHM group (*P* > 0.05), suggesting that DHM chemotherapy was non-toxic towards blood samples. Our results from *in vivo* tests preliminarily demonstrated that DHM chemotherapy could significantly suppress MIBC tumorigenesis while trigger no obvious drug toxicity. This study has screened out a plant-derived drug, which was desirable for the application of MIBC chemotherapy.

## Discussion

Original advances in the biomedical fields, especially innovative drug, technology and equipment, have greatly improved the existing clinical diagnosis and treatment systems (Wang G. Y. et al., 2024; Hu et al., 2024). For instance, the use of chemotherapeutic agents has notably extended the survival rates of cancer patients. Presently, the chemotherapeutic agents available predominantly include platinum compounds, hormones, antibodies, alkylating agents, antimetabolites, and plant-derived drugs, among others (Bojko et al., 2019). Particularly, some agents like paclitaxel, cisplatin and gemcitabine, have been widely used in clinic (Bojko et al., 2019; Hu et al., 2019). MIBC is a malignant tumor prone to recurrence and metastasis, and the effect of post-operative chemotherapy of MIBC is far from satisfactory. Some methods like combination chemotherapy, neoadjuvant chemotherapy and bladder irrigation, have been developed to solve this problem (Li et al., 2024). It is promising to explore more candidate agents with excellent anti-tumor activity and biosafety for MIBC chemotherapy.

Dihydromyricetin (DHM) is a natural flavonoid which was extracted from the Chinese herbal medicine known as ratten tea. Similar with the other PPDs, DHM demonstrates significant advantages in terms of biocompatibility and non-toxicity. Furthermore, DHM exhibits potent anti-tumor activity against various cancers, including lung cancer, ovarian cancer, colorectal cancer, and cutaneous squamous cell carcinoma. However, the *in vitro* and *in vivo* applications of DHM for MIBC chemotherapy have rarely been reported. In this study, we firstly found that DHM could inhibit the proliferation and migration of MIBC (T24 and UMUC3) cells, and promote cell apoptosis. Interestingly, these anti-tumor properties of DHM were modulated in a dose-dependent manner (Figures 1a,b). The findings from *in vivo* experiments further demonstrated that DHM effectively inhibited the growth of xenografts without inducing significant toxicity (Figure 6). Consequently, DHM emerges as a promising candidate for chemotherapy in MIBC. Nonetheless, the molecular mechanisms underlying its action and its biosafety profile require further elucidation prior to clinical application.

The anti-tumor mechanisms of DHM exhibit significant variability across different cancer types. For instance, research conducted by Zhiqiang Zhao and colleagues has demonstrated that DHM can inhibit the tumorigenesis of osteosarcoma by modulating the P38 (MAPK) and AMPKα/GSK3β/Sox2 signaling pathways (Zhao et al., 2014). Another literature from Bin Liu have reported that DHM could induce hepatocellular carcinoma cell apoptosis, and further slowed production of reactive oxygen species (ROS) (Liu et al., 2015). In general, the anti-tumor mechanism of DHM may be involved into the following biological processes, such as cell cycle arrest, apoptosis, ferroptosis, autophagy, ROS production, EMT and angiogenesis inhibition, et al. (Tan et al., 2019). Our research team conducted a screening of the differentially expressed genes between carcinoma tissue and adjacent non-carcinoma tissue in MIBC (Ge et al., 2019; Zhou et al., 2019). Subsequently, we validated the function and mechanism of the candidate genes. Our findings indicate a strong correlation between cell cycle regulation, apoptosis, and EMT with the development and progression of MIBC. In this study, we have identified the expression of key hub genes or markers involved in the processes of cell cycle arrest, apoptosis, and EMT. It was observed that the cell cycle and apoptosis signaling pathways were both interrupted by DHM treatment (Figures 2, 3), and the EMT in BCa cells was successfully inhibited (Figure 4). Other unknown mechanisms of DHM will be investigated in the future work.

Biosafety evaluation is one of the core components in the preclinical study of pharmaceutical preparations, nanomaterials and implantable devices. Zhang et al. have proposed a widely utilized histological method for the assessment of drug toxicity. Briefly, the treated animals were executed, and the organs like heart, liver, spleen, lung, kidney and brain were dissected for histological analysis. In this work, the tumor bearing assay and *in vivo* biosafety evaluations were performed at the same time. Intravenous injection of DHM with a dose of 20 mg/kg for 7 times successfully suppressed the xenograft growth, but led to potential organ toxicity to lung tissues (Figure 6f). In order to minimize the side effects, the dose and frequency of DHM injection in the animal experiments should be cut down. On the other hand, DHM could be modified and functionalized by biomaterials science and technology. For example, DHM could be encapsulated into tumor cell derived exosomes, and then targeted to cancer cells and kill them precisely (Liu et al., 2018; Liu W. L. et al., 2019). A large number of tumor nano-platforms, such as exosome, cell membrane vesicle, metal-organic framework (MOF),carbon nanotube (CNT) and microfluidic, has made it possible for precision medicine (Kim et al., 2019; Gao et al., 2019; Wang et al., 2018; Chen et al., 2019; Wang et al., 2025).

## Data Availability

The raw data supporting the conclusions of this article will be made available by the authors, without undue reservation.
