# Supplementary material for: Chemotherapeutic dihydromyricetin with remarkable anti-tumor activity and biosafety for muscle invasive bladder cancer
Source: Front Pharmacol. 2025 Jul 18;16:1609354. doi: 10.3389/fphar.2025.1609354 (PMC12314755; doi:10.3389/fphar.2025.1609354)
Supplement: Supplementary file 1 [file DataSheet1.docx]

**Characterizations of chemotherapeutic dihydromyricetin with enhanced anti-tumor activity and biosafety for muscle invasive bladder cancer**

Zicheng Guo^a,b,1^, Wang Wang^a,1^, Weikang Hu^d^, Wenjie You^c,*^, and Zijian Wang^b,^[[1]](#footnote-1)^*^

^a^ Department of Urology, The Central Hospital of Enshi Tujia and Miao Autonomous Prefecture, Enshi 445000, P. R. China

^b^ Department of Urology, Hubei Key Laboratory of Urological Diseases, Cancer Precision Diagnosis and Treatment and Translational Medicine Hubei Engineering Research Center, Zhongnan Hospital of Wuhan University, Wuhan 430071, China

^c^ Orthopedic Hospital, Postdoctoral Innovation Practice Base, The First Affiliated Hospital, Jiangxi Medical College, Nanchang University, Nanchang, 330006, China

^d^ Ministry of Education Key Laboratory of the Green Preparation and Application for Functional Materials, Hubei Key Laboratory of Polymer Materials, School of Materials Science and Engineering, Hubei University, Wuhan 430062, China

**
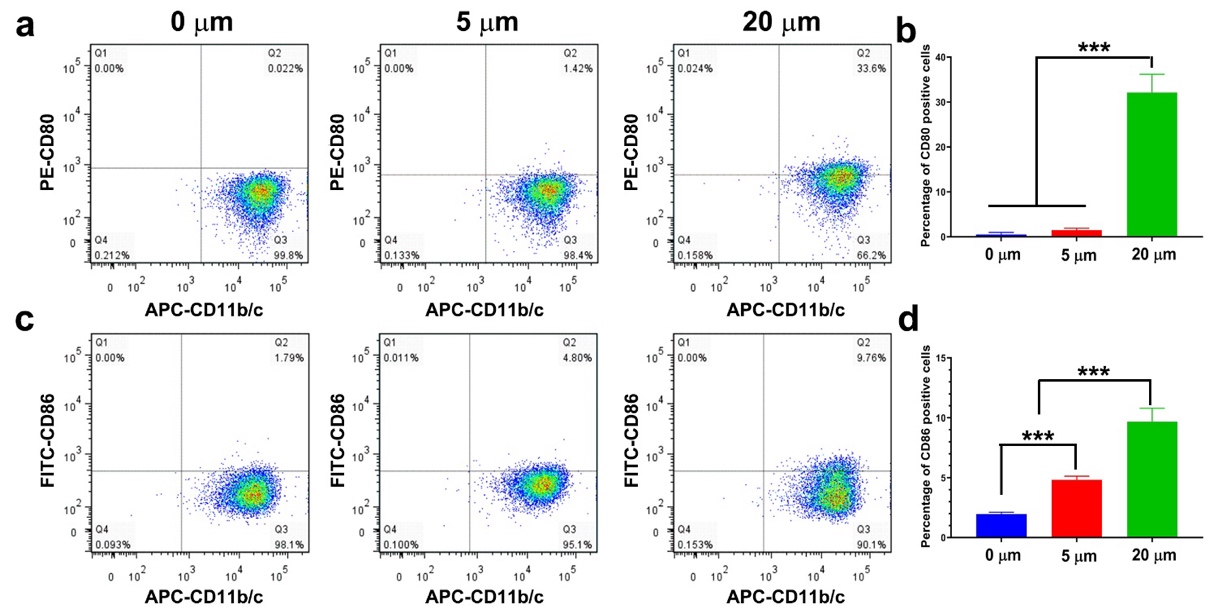
**

**Figure S1.** Dihydromyricetin (DHM) motivated macrophage polarization in vitro. (a, c) Representative flow cytometry images of CD80 and CD86 staining after DHM chemotherapy; (b, d) Quantitative results of CD80 and CD86 positively stained cells. ****P* < 0.001.

**
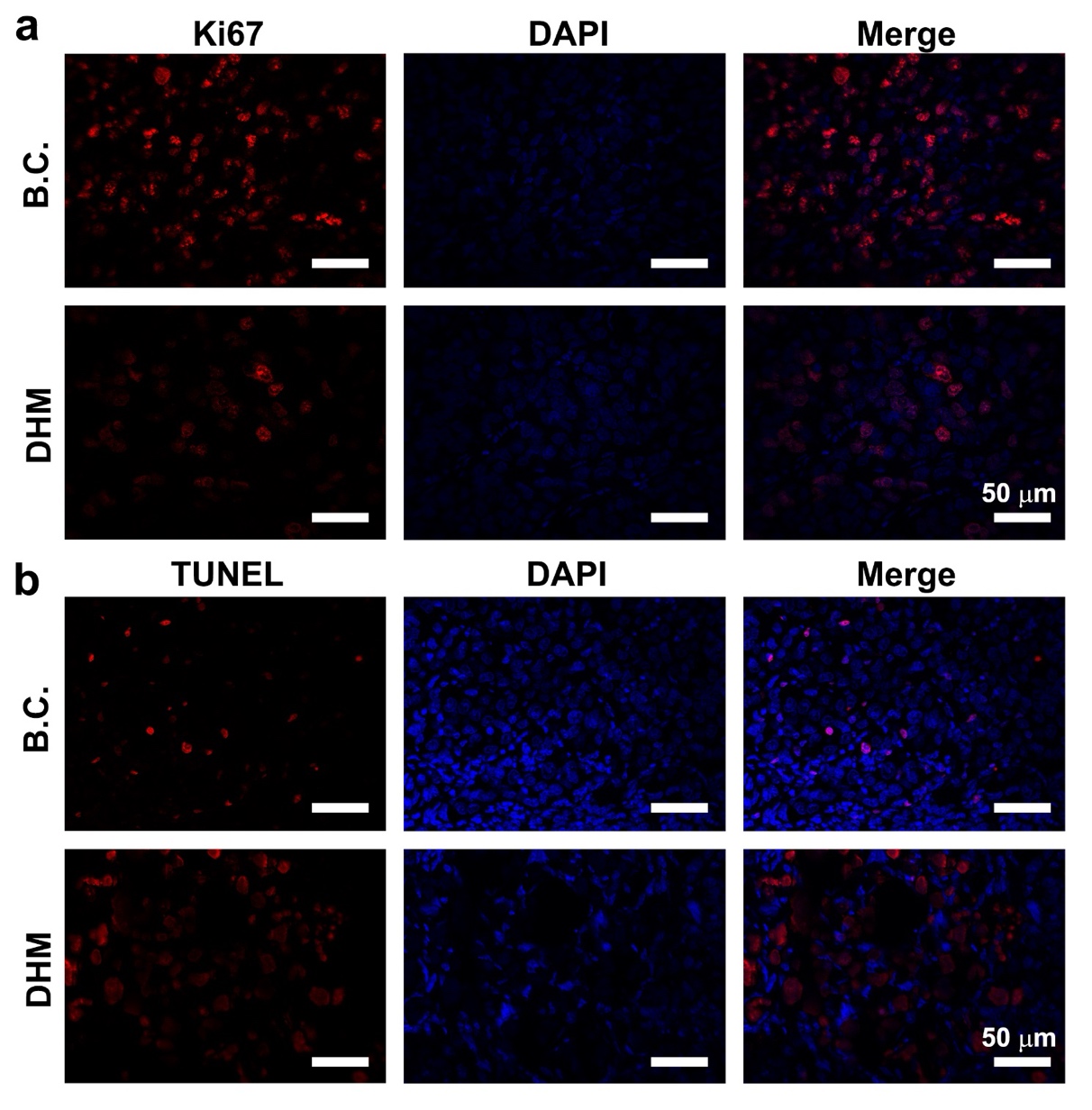
**

**Figure S2.** Dihydromyricetin (DHM) inhibited proliferation and promoted apoptosis of BCa in vivo xenografts. (a) Representative Ki67 staining images of the tumor samples. Scale Bar: 50 μm. (b) Representative TUNEL staining images of the tumor samples. Scale Bar: 50 μm.

**
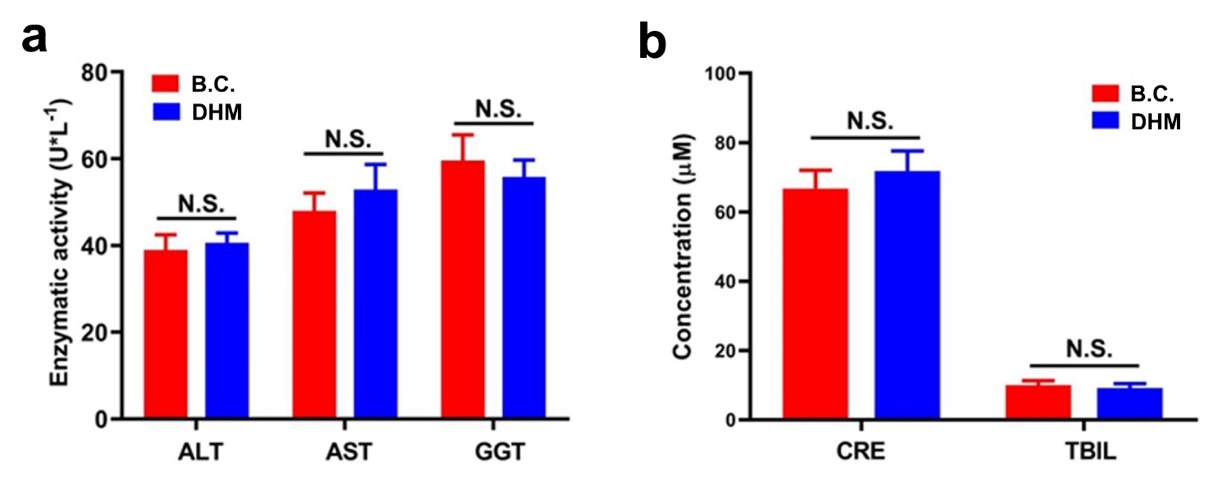
**

**Figure S3.** Dihydromyricetin (DHM) possessed remarkable in vivo hemocompatibility. (a) Quantitative results of enzymatic activity of ALT, AST and GGT. (b) Representative results of the concentration of CRE and TBIL. N.S. represented *P* > 0.05.


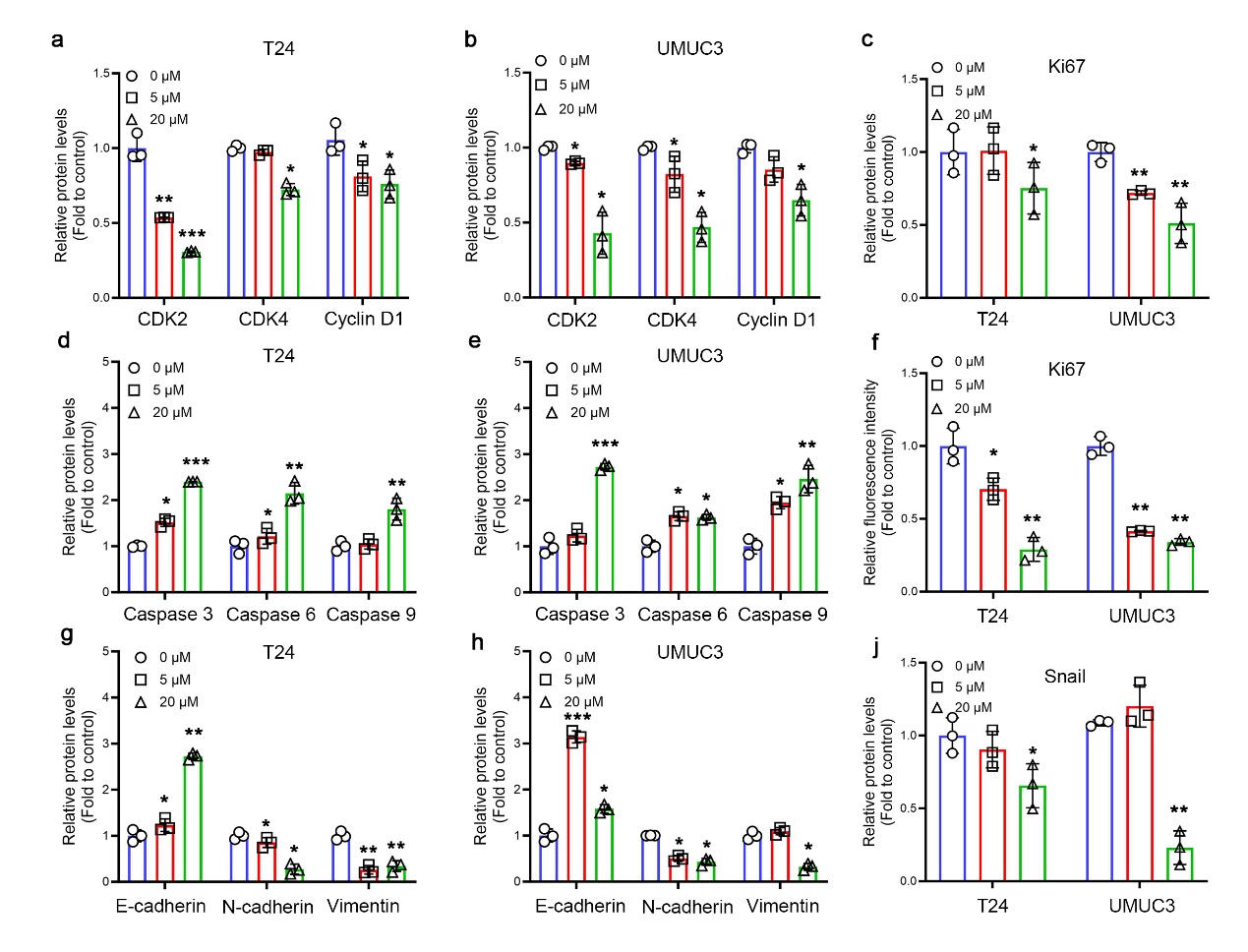


**Figure S4.** (a) The West-blot quantitative analysis results of CDK2, CDK4, Cyclin D1 in T24. (b) The West-blot quantitative analysis results of CDK2, CDK4, Cyclin D1 in

UMUC3. (c) The West-blot quantitative analysis results of Ki67. (d) The West-blot quantitative analysis results of Caspase 3, Caspase 6, Caspase 9 in T24. (e) The West-blot quantitative analysis results of Caspase 3, Caspase 6, Caspase 9 in UMUC3. (f) The immunofluorescence staining quantitative analysis results of Ki67. (g, h, j) The West-blot quantitative analysis results of E-cadherin, N-cadherin, Vimentin, Snail. **P* < 0.05, ***P* < 0.01, ****P* < 0.001.

**Table S1.** Primer sequences used for qRT-PCR

| Primer name | sequences 5’-3’ | Tm |
| --- | --- | --- |
| GAPDH-F | TGTGGGCATCAATGGATTTGG | 60.9 ºC |
| GAPDH-R | ACACCATGTATTCCGGGTCAAT | 61.4 ºC |
| CDK2-F | TGTTTAACGACTTTGGACCGC | 61.1 ºC |
| CDK2-R | CCATCTCCTCTATGACTGACAGC | 61.5 ºC |
| CDK4-F | GGGGACCTAGAGCAACTTACT | 60.3 ºC |
| CDK4-R | CAGCGCAGTCCTTCCAAAT | 60.4 ºC |
| Cyclin E1-F | TCGCATCAAACTCTCTGGCTA | 60.9 ºC |
| Cyclin E1-R | TGAGCGACTAAACTCACCACT | 60.8 ºC |
| Cyclin D1-F | GCTGCGAAGTGGAAACCATC | 61.6 ºC |
| Cyclin D1-R | CCTCCTTCTGCACACATTTGAA | 60.8 ºC |
| P53-F | AACTGCGGGACGAGACAGA | 62.9 ºC |
| P53-R | AGCTTCAAGAGCGACAAGTTTT | 60.7 ºC |
| Vimentin-F | GCCCTAGACGAACTGGGTC | 61.4 ºC |
| Vimentin-R | GGCTGCAACTGCCTAATGAG | 61.1 ºC |
| E-cadherin-F | CGAGAGCTACACGTTCACGG | 62.6 ºC |
| E-cadherin-R | GGGTGTCGAGGGAAAAATAGG | 60.1 ºC |
| N-cadherin-F | TTTGATGGAGGTCTCCTAACACC | 61.3 ºC |
| N-cadherin-R | ACGTTTAACACGTTGGAAATGTG | 60.2 ºC |
| Snail-F | TGTGACAAGGAATATGTGAGCC | 60.0 ºC |
| Snail-R | TGAGCCCTCAGATTTGACCT | 61.5 ºC |
| Caspase 3-F | AGAGGGGATCGTTGTAGAAGTC | 60.6℃ |
| Caspase 3-R | ACAGTCCAGTTCTGTACCACG | 61.4℃ |
| Caspase 6-F | GAGCACGTTGGATATGATGGTG | 61.1℃ |
| Caspase 6-R | GGTCCACTTACATCCTCGATCTA | 60.4℃ |
| Caspase 9-F | ATGTCGGACTACGAGAACGAT | 60.4℃ |
| Caspase 9-R | TGATGCGTGAGGGGTCGAT | 63.0℃ |

**Table S2.** List of primary antibodies and secondary antibodies

| name | species | dilution | resources |
| --- | --- | --- | --- |
| GAPDH | Mouse | 1:1000 | Abcam, USA, Cat. # ab181602 |
| CDK2 | Rabbit | 1:1000 | Abcam, USA, Cat. # ab32147 |
| CDK4 | Rabbit | 1:1000 | Abcam, USA, Cat. #ab108357 |
| Cyclin D1 | Rabbit | 1:1000 | Abcam, USA, Cat. #ab16663 |
| Ki67 | Rabbit | 1:100 | Abcam, USA, Cat. # ab15580 |
| E-cadherin | Rabbit | 1:1000 | Abcam, USA, Cat. #ab194982 |
| N-cadherin | Rabbit | 1:1000 | Abcam, USA, Cat. #ab18203 |
| Snail | Rabbit | 1:1000 | Abcam, USA, Cat. #ab229701 |
| Vimentin | Rabbit | 1:1000 | Abcam, USA, Cat. #ab93547 |
| Caspase 3 | Rabbit | 1:1000 | Abcam, USA, Cat. #ab51772 |
| Caspase 6 | Rabbit | 1:1000 | Abcam, USA, Cat. #ab32053 |
| Caspase 9 | Rabbit | 1:1000 | Abcam, USA, Cat. #ab219590 |
| Anti-Mouse-IgG (H+L)-HRP | Goat | 1:10000 | Sungene Biotech, China, Cat. #LK2003 |
| Anti-Rabbit-IgG(H+L)-HRP | Goat | 1:10000 | Sungene Biotech, China, Cat. #LK2001 |

1. *Corresponding authors at:

   Department of Urology, Zhongnan Hospital of Wuhan University, Wuhan 430071, China. E-mail address: [Zijianwang@whu.edu.cn](mailto:Zijianwang@whu.edu.cn) (Z. Wang); Orthopedic Hospital, The First Affiliated Hospital, Jiangxi Medical College, Nanchang University, Nanchang, 330006, China. E-mail address: [youwenjie@ncu.edu.cn](mailto:youwenjie@ncu.edu.cn) (W. You)

   ^1^ These authors contributed equally to this work. [↑](#footnote-ref-1)
